# Reconstruction of *apo* A2A receptor activation pathways reveal ligand-competent intermediates and state-dependent cholesterol hotspots

**DOI:** 10.1038/s41598-019-50752-6

**Published:** 2019-10-02

**Authors:** Silvia Lovera, Alberto Cuzzolin, Sebastian Kelm, Gianni De Fabritiis, Zara A. Sands

**Affiliations:** 1 CADD, UCB BioPharma, 1420 Braine l’Alleud, Belgium; 2 Acellera, Barcelona Biomedical Research Park (PRBB), C/Doctor Aiguader 88, 08003 Barcelona, Spain; 3 CADD, UCB Pharma, Slough, UK; 4 Computational Science Laboratory (GRIB-IMIM), Universitat Pompeu Fabra, Barcelona Biomedical Research Park (PRBB), C/Doctor Aiguader 88, 08003 Barcelona, Spain; 5 Institució Catalana de Recerca i Estudis Avançats (ICREA), Passeig Lluis Companys 23, 08010 Barcelona, Spain

**Keywords:** Computational biophysics, Molecular modelling

## Abstract

G-protein coupled receptors (GPCRs) play a pivotal role in transmitting signals at the cellular level. Structural insights can be exploited to support GPCR structure-based drug discovery endeavours. Despite advances in GPCR crystallography, active state structures are scarce. Molecular dynamics (MD) simulations have been used to explore the conformational landscape of GPCRs. Efforts have been made to retrieve active state conformations starting from inactive structures, however to date this has not been possible without using an energy bias. Here, we reconstruct the activation pathways of the *apo* adenosine receptor (A2A), starting from an inactive conformation, by applying adaptive sampling MD combined with a goal-oriented scoring function. The reconstructed pathways reconcile well with experiments and help deepen our understanding of A2A regulatory mechanisms. Exploration of the *apo* conformational landscape of A2A reveals the existence of ligand-competent states, active intermediates and state-dependent cholesterol hotspots of relevance for drug discovery. To the best of our knowledge this is the first time an activation process has been elucidated for a GPCR starting from an inactive structure only, using a non-biased MD approach, opening avenues for the study of ligand binding to elusive yet pharmacologically relevant GPCR states.

## Introduction

G-protein coupled receptors (GPCRs) play a pivotal role in transmitting signals at the cellular level. Their deregulation is often associated with pathological conditions, thus making them major therapeutic targets^1,2^. One such protein, the adenosine receptor (A2A), belonging to the class A subfamily, has been implicated in diseases such as cardiovascular disorders and Parkinson’s disease^3^.

Structural determination of GPCRs remains challenging due to their intrinsic flexibility. However, thanks to recent advances in structural biology for membrane proteins, dozens of A2A crystal structures have been solved, increasing our understanding of the complex link between structure and function^4^. For example, a single GPCR can couple with multiple G proteins, as well as β-arrestin, triggering different signalling pathways^5^. This evidence does not support the simplistic Katz two-state model^6^, but rather the hypothesis that an ensemble of multiple active and inactive conformations coexist and account for the promiscuous coupling of these receptors^7,8^.

Receptor modulation is mainly achieved by the binding of ligands, and even membrane lipids, by changing the relative populations of active versus inactive conformations^9–11^. This is also true for the A2A receptor. Growing experimental^12,13^ and computational evidence^14–16^ suggest that A2A can adopt metastable intermediates upon activation. However, the nature and role played by these intermediates is not fully understood. In this context, the study of the conformational energy landscape is useful to understand the structural changes that culminate in GPCR activation. Molecular Dynamics (MD) is typically used to study protein flexibility and transitions among different conformational states, which are not always observable in biophysical experiments^17–19^.

Reconstructing the activation pathway of a GPCR using classical MD is challenging, due the high energetic barrier for activation (cf. 12 kcal/mol for M2 receptor)^20^. Usually the pathway is recovered by allowing the *apo* form of the agonist-bound structure to progress towards a lower-energy inactive state^14,16,21–23^. Enhanced sampling methods have also been extensively applied to GPCRs^20,22–24^. However, the use of an energetic bias could lead to artifacts.

Here we reconstruct in an unbiased manner the activation pathway(s) of the A2A receptor by applying Markov State Modelling (MSM) to the analysis of MD simulations^25,26^ generated using adaptive sampling combined with a goal-oriented scoring function^27^. This method is similar to other unbiased techniques such as Supervised MD^28,29^ and FAST^30^. Starting from the *apo* A2A inactive structure, we were able to recover the active conformation and identify ligand-competent states. Moreover, analysis of the results by MSM helped us to elucidate alternative activation pathways enhancing our understanding of the inner mechanisms that regulate receptor activation.

## Results

### Adaptive sampling allows the fast reconstruction of the *apo* A2A activation landscape without a priori structural information

**Figure 1:**
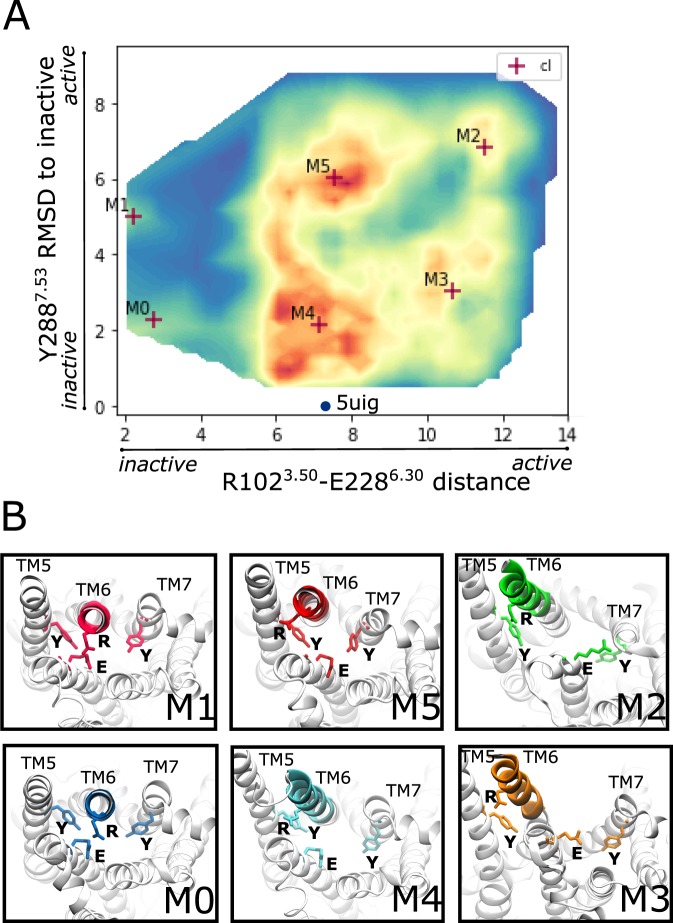
*Apo* A2A activation landscape. (**A**) Density map of the *apo* A2A receptor structures sampled during the adaptive simulation plotted along the two descriptors characterising GPCR activation: the distance between R102^3.50^ and E228^6.30^ and the RMSD from the inactive position of residue Y288^7.53^. The blue dot represents the crystal structure 5uig that was used as starting structure in the MD simulation. Cluster centroids (cl) of the six kinetic macrostates are projected with red markers and the numbering is assigned from the least populated to the more populated cluster. (**B**) Structures corresponding to the six macrostates identified by the MSM model. Specific residues and structural elements whose change in conformation characterize active, inactive and intermediates states in A2A are highlighted (pink, blue, red, cyan, green and orange for M1, M0, M5, M4, M2 and M3 respectively). These features include: the TM6 helix, the ionic lock residues (R102^3.50^ and E228^6.30^), Y197^5.58^ of TM5 and Y288^7.53^ of TM7.

We reconstructed the activation landscape of the *apo* A2A receptor, starting from the inactive crystallographic structure (pdb code 5uig)^31^ after equilibration (see Material and Methods). Using adaptive sampling in combination with a goal-oriented scoring function^32^ we were able to exhaustively sample the conformational space of the receptor. The adaptive simulation was set up in such a way that a generic metric, which considers α-Cα protein contact maps, was coupled with a specific goal-oriented function. The variables considered in the ‘goal’ are known structural elements of GPCRs that differentiate inactive and active conformations^14,21^, namely: the distance between residues R102^3.50^ and E228^6.30^ (a.k.a. ionic lock), and the RMSD of residue Y288^7.53^ in the inactive crystal structure (5uig.pdb) (the superscript of the residues used throughout the paper refers to the Ballesteros−Weinstein numbering for GPCRs). The first set of residues characterize a salt bridge that is observed in the majority of GPCR inactive crystal structures. The salt bridge is lost when the receptor is activated, due to the outward movement of TM6. Y288^7.53^ belongs to the highly conserved NPXXY motif and is involved in the inward movement of TM7 upon activation. Projection of the trajectories along the two activation variables (Fig. 1, panel A) clearly shows that the inactive crystal structure of *apo* A2A has successfully explored a vast conformational landscape, in which several metastable states could be identified, including inactive and active intermediates. A converged MSM model of the *apo* A2A receptor was recovered in only 80 µs of aggregated simulation time (see Table S1 in SI Appendix). The conformational space was subsequently discretized into 890 clusters. The resulting MSM model was constructed at 20 ns lag time and clusters were grouped into 6 macrostates (see Material and Methods and Fig. S1 in SI Appendix for details). Centroids of the six kinetic macrostates are plotted in Fig. 1, panel A.

Based on the obtained equilibrium distribution (Fig. S2 in SI Appendix), the majority of the population belong to macrostates M5 and M4 (38.4% ± 2.3 and 31.9% ± 1.5, respectively). Macrostate M4 is characterized by an ensemble of inactive conformations, whereas macrostate M5 adopts conformations close to known agonist-bound x-ray structures. Macrostates M3 and M2 are the next most densely-populated, with percentages of 17.8% ± 1.3 and 9.9% ± 1.1 respectively. The least-populated macrostates are M1 and M0 with very low percentages, 1.2% ± 0.4 and 0.6% ± 0.2 respectively. By analysing the ensemble of conformations belonging to each macrostate, recognized structural elements were used to identify active, inactive and intermediate states (Fig. 1, panel B). More specifically, the inactive M0 and M4 macrostates are characterized by TM3-TM6 distances of 2 and 6 Å, meaning that the ionic lock interaction is maintained in M0 and broken in M4. In M4 the TM3-TM6 distance fluctuates between 6 and 8.5 Å. This enlarged inactive basin has been also described in the work of Caliman *et al*.^14^. The most populous M5 has a shorter TM3-TM6 distance (6–8.5 Å) indicative of a GPCR inactive state, while the conformation of TM7 is characteristic of an active one (with high RMSD values of Y288^7.53^ compared to the inactive). Therefore, overall M5 could be considered as an A2A active-like intermediate. Macrostate M1 is close in conformational space to M5 and is the least populated state. M1 is differentiated from M5 through the formation of the ionic lock between R102^3.50^ and E228^6.30^ (Fig. 1, panel B). The other recognizable intermediate in the activation landscape is M3. This macrostate, unlike M5, is characterized by a longer TM3-TM6 distance (from 9.5 to 11Å) and an inactive-like conformation of Y288^7.53^, that is mostly seen to loosely interact with residue N284^7.49^. This finding is in agreement with the intermediate conformation also identified by Caliman *et al*.^14^. Finally, the A2A landscape includes the metastable macrostate M2, recognized to be the closest to the solved active A2A miniG_s_-bound structures (pdb codes 5g53^33^ and 6gdg^34^). This macrostate shows the largest TM3-TM6 distance, reaching 12Å, which is also in agreement with the active state reported in Caliman *et al*.^14^.

### MSM identifies ligand-competent intermediate states in the *apo* conformational landscape of A2A

**Figure 2:**
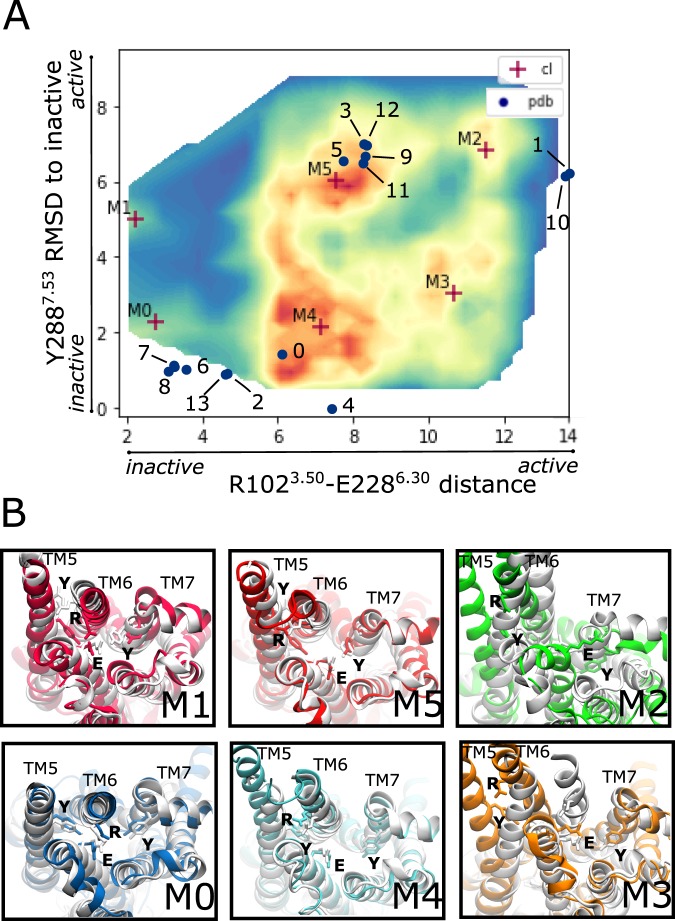
Ligand-competent intermediate states. (**A**) Density map of the *apo* A2A receptor plotted along the two descriptors of GPCR activation and sampled during simulation: the distance between R102^3.50^ and E228^6.30^ and the RMSD from inactive of residue Y288^7.53^. The blue dots represent the distances of some of the solved crystal structures of A2A in the Protein Data Bank: 0 = 3eml, 1 = 5g53, 2 = 4eiy, 3 = 2ydv, 4 = 5uig, 5 = 3qak, 6 = 5nm2, 7 = 3pwh, 8 = 3rfm, 9 = 4ug2, 10 = 6gdg, 11 = 5wf5, 12 = 2ydo, 13 = 5olg (see Table S2 in Supplementary Information for details on the considered crystals). Centroids of the six kinetic macrostates have been projected and identified by red markers. (**B**) Structures corresponding to the six macrostates identified by the MSM model are differently coloured (pink, blue, red, cyan, green and orange for M1, M0, M5, M4, M2 and M3 respectively) and aligned to the Cα atoms of the following crystal structures: M1, M5 aligned with agonist-bound 2ydo; M2 aligned with miniG_s_-protein bound 5g53; M0 aligned with antagonist-bound 3pwh, M4, M3 aligned with antagonist-bound 3eml.

There are many similarities between our macrostates and the solved A2A crystal structures. In Fig. 2, a representative group of A2A crystals are plotted onto the reference landscape of the simulated *apo* A2A. As expected, agonist, antagonist and miniG_s_-bound structures cluster in different and very specific areas of the conformational landscape. For example, antagonist-bound structures cluster near to the inactive macrostates M0 and M4 (bottom left of Fig. 2, panel A). Indeed, M0 and M4 structures resemble the reference antagonist-bound crystals 3pwh^35^ and 3eml^36^, respectively. As shown in Fig. 2 panel B, the ionic lock is formed in M0 as in structure 3pwh and broken in M4 as in 3eml. In the *apo* structure, macrostate M4 is more populated than M0. In contrast, four out of seven of the available antagonist-bound crystal structures have a TM3-TM6 distance between 2 and 4 Å, resembling M0. Thus, it may appear that antagonists may prevalently select for a conformation with a formed ionic lock while, the *apo* inactive state (M4) would prefer a broken ionic-lock. This observation is in agreement with a previous study by Li *et al*. Indeed, the study showed that when no ligand is bound, the TM6 helix of the A2A receptor adopts a separate conformation, between the inactive antagonist-bound and the active state^16^. Thus, it appears that the *apo* receptor maintains a substantial part of its population in an ‘intermediate’, M4, inactive state.

The agonist-bound crystal structures cluster in a defined region of the 2D plot corresponding to the most populated macrostate M5 (upper part of the plot, see Fig. 2 panel A). All solved agonist-bound structures are structurally very similar. Each of them is missing some of the features characteristic of the active receptor, such as the outward movement of TM6, and a change in the rotamers of TM7 with Y288^7.53^ pointing towards the TM5 helix, thus making the G-protein binding site inaccessible. It has been observed that agonists stabilize a state that is not fully G-protein competent but instead represents an active intermediate of the A2A receptor^33,37^. Alignment of the agonist-bound reference structure 2ydo^37^ to the representative M5 structure reveals that they are strikingly similar (see Fig. 2, panel B). This leads us to conclude that the agonist-bound crystal structures do indeed represent an active intermediate. Thus, in our reconstruction of the *apo* A2A landscape, M5 represents the agonist-competent state.

As shown in Fig. 2, the active G-protein bound crystal structures (points 1 and 10 in panel A) appear in a poorly-explored area, close to M2. From our calculations we observe that the G-protein bound state is scarcely populated. Evidence from both wet and *in silico* experiments may help to explain this observation. For example, the work of Murphree *et al*. revealed how the G-protein has a higher affinity for the receptor when an agonist is bound compared to the *apo* form^38^. By comparing macrostate M2 and the miniG_s_-bound structure, M2 has a TM3-TM6 distance that only reaches 12 Å, compared to the 14 Å seen in 5g53 and 6gdg crystal structures, but its TM5 is more open. Since the TM3-TM6 distance alone might not be enough to describe the overall opening of the G-protein binding site, distances of all TM pairs were calculated for the intracellular portion of the receptor and summed. The average summed distances for the inactive (M4) and active-like (M2) macrostates were compared to the distance of the miniG_s_-bound crystal structure 5g53 (see Material and Methods and Fig. S3 in SI Appendix). Summed distance values for the G-protein binding site of 5g53 is 286.2 Å, while the average for M4 and M2 states are 279.5 ± 13.08 Å and 330.5 ± 11.95 Å, respectively. It appears that the intracellular portion of macrostate M2 may easily accommodate the G-protein, even in the absence of an agonist. The existence of this small population of the *apo* receptor (approximately 10%), able to bind the G-protein, reconcile well with the concept of receptor basal activity^39^, identifying M2 as the G-protein competent state.

### More than one activation pathway is possible for the *apo* A2A receptor

**Figure 3:**
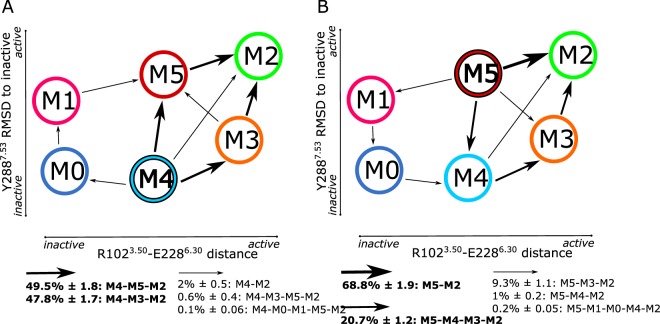
Activation pathways of the apo A2A receptor. Each macrostate is schematically represented by a coloured labelled circle mapped on the two descriptors of A2A activation. Percentages for each pathway flux are reported below the corresponding plot with the corresponding standard deviation. The thickness of the arrows correlates with the value of the related percentage. The thicker the arrow, the higher the value, and thus, the relevance of the pathway observed. (**A**) Net kinetic flux reconstructed from the MSM model built for the *apo* A2A receptor. The reconstructed activation pathway considers the transition from M4 (inactive-like state) to M2 (active-like state). (**B**) Net kinetic flux reconstructed from the MSM model built for the *apo* A2A receptor. The reconstructed activation pathway considers the transition from M5 (active intermediate) to M2 (active-like state).

Transition path theory (TPT) was used to recover the kinetic pathway among the identified macrostates. The pathway flux may be considered as the number of times a structure A moves to B via a certain pathway during the considered lag time. Given the metric used to build the MSM model, only a qualitative analysis of the pathways could be described. This is a consequence of the implied timescales obtained by considering the two activation variables not accounting for the receptor slowest motions. The result is an underestimation of the kinetics of activation. Considering macrostate M4 as the A2A inactive-like state and M2 as the G-protein competent state, the fully connected activation pathway analysis identifies two main routes with almost equal probabilities (Fig. 3, panel A). The first is via the intermediate agonist-bound M5 state, and the second is *via* the M3 intermediate. These two pathways account for 49.5% ± 1.8 and 47.8% ± 1.7 of the total explored activation pathway, respectively. From a structural perspective, it means the A2A structure would be slightly more prone to undergo activation by first rotating the intracellular part of TM7 helix inward, followed by the outward movement of the TM6 helix. The remaining 3% of the pathway reveals a strong interconnection of M4 with the other macrostates, with M4 essentially acting as a “conformational hub” in the *apo* landscape. Since M5 is the most populated macrostate and the one most favoured by agonists, the pathway from M5 to M2 was also reconstructed (see Fig. 3, panel B). A structure belonging to M5 would preferentially shift directly towards the G-protein competent M2 state (68.8% ± 1.9 of the entire pathway). M5 would rarely shift to M2 via M4 and M3 (20,7% ± 1.2).

### Is Cholesterol an additional player in A2A activation?

**Figure 4:**
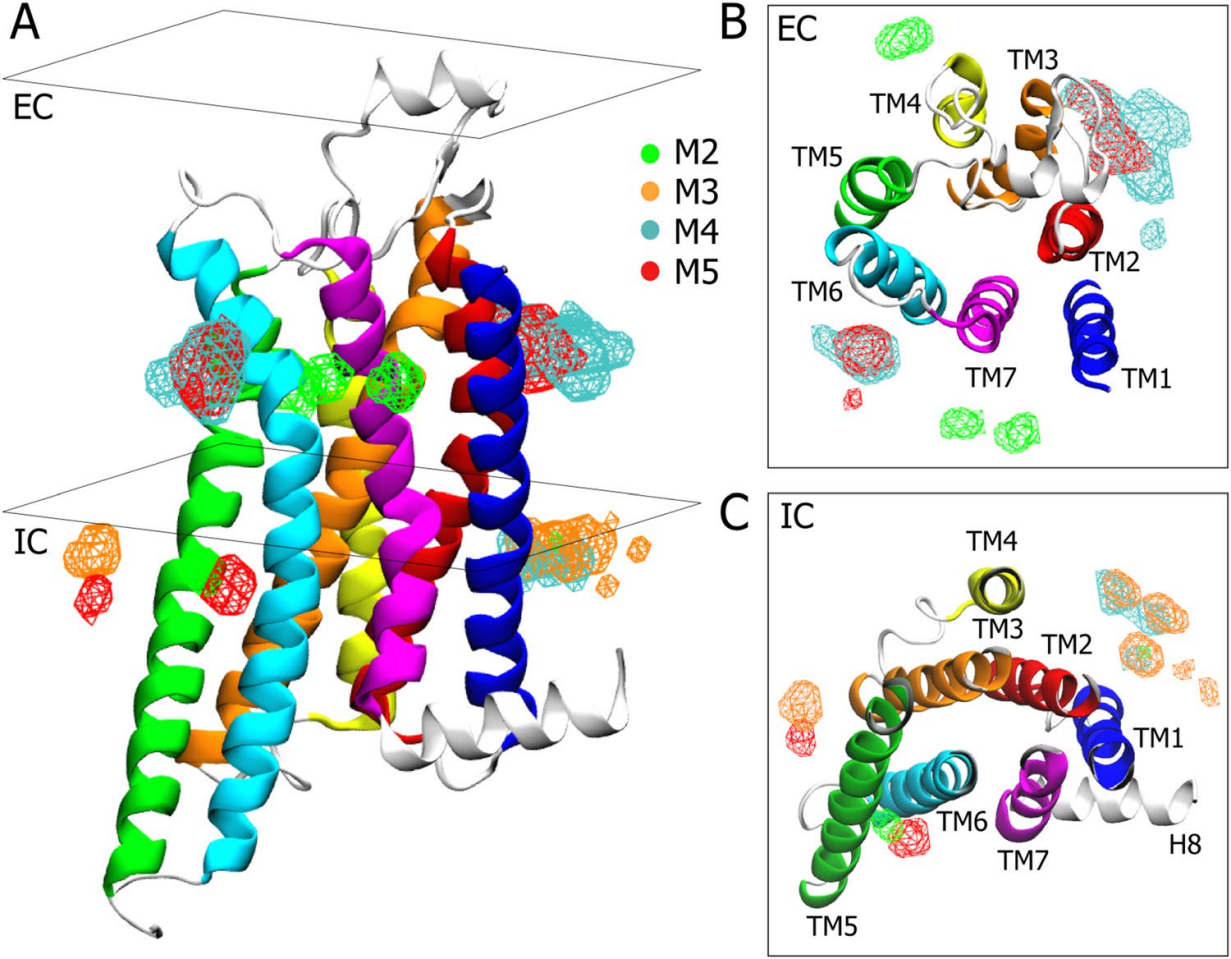
Cholesterol hotspots. Cartoon representation of the A2A receptor showing cholesterol occupancy for each of the most populated macrostates: M2, M3, M4 and M5. (**A**) Mesh surfaces represent the hotspots where cholesterol has the higher occupancy. Each surface is colour-coded to correspond to the respective macrostate. (**B**) Transverse sectional view of the EC aspect of the A2A receptor. The hotspots corresponding to M2, M4 and M5 are shown in green, cyan and red respectively. (**C**) Transverse sectional view of the IC aspect of the A2A receptor. The hotspots corresponding to M2, M3, M4 and M5 are shown in green, orange, cyan and red respectively.

Cholesterol is known to play a key role in membrane structure and GPCR regulation^40–42^. Although cholesterol binding sites have been reported for many GPCRs, including A2A^43–46^, its effect on receptor activation is still unclear. In this study we simulated the *apo* A2A receptor in a POPC membrane with 20% cholesterol. We calculated occupancy of cholesterol and identified high-occupancy hotspots for the most-populated macrostates M2, M3, M4 and M5 (see details in Material and Methods). Interestingly, these hotspots are not maintained throughout all of the macrostates but change depending on the state of the receptor. For example, the ones identified for the inactive M4 macrostate (cyan mesh surface in Fig. 4) overlap with those shown in the 4eiy and 5iu4 inactive crystal structures^47,48^. These are located at the extracellular (EC) cleft between TM2-TM3 and the EC portion of TM6. In addition, another hotspot was identified towards the intracellular aspect (IC) of the receptor between TM1 and TM2 helices. We identified unique cholesterol hotspots in the EC region of TM4 and in between TM1-TM7 helices for the active G-protein competent state, M2 (green mesh surface in Fig. 4), whilst revealing some others in the IC region of TM1 and TM6 in common with M3 and M5 respectively. Interestingly, the intermediate states M5 and M3 partially share those of M4 and M2 (see Fig. 4).

**Figure 5:**
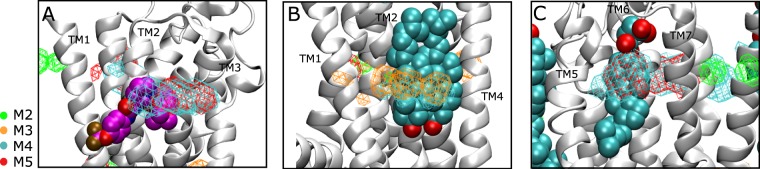
Allosteric compounds and lipids binding sites. Examples of allosteric compound and lipids binding to areas of the A2A receptor with high cholesterol occupancy. (**A**) Structure of BPTU allosteric compound (in magenta) bound to the TM2-TM3 EC cleft of P2Y1 (pdb code 4xnv). (**B**) Two molecules of cholesterol bound to the TM2-TM3-TM4 IC cleft of β2 adrenoreceptor (pdb code 5x7d). (**C**) Cholesterol hemisuccinate bound to TM6-TM7 EC cleft of P2Y1 (pdb code 4xnv).

Since no molecules of cholesterol have been co-crystallized in any G-protein or agonist-bound crystal structure, we were not able to directly compare our results, as we did for M4. However, our results are to some extent in agreement with other computational studies, wherein cholesterol hotspots were found for A2A in the TM2-TM3, TM1-TM7 and TM3-TM5 clefts^45,46,49,50^. Moreover, in Fig. 5 we report some examples in which the identified densities match co-crystallized allosteric compounds or lipids in homologous structures. Overall, cholesterol appears to interact with the *apo* A2A receptor via transient sites that dynamically change in a state-dependent manner. Some of these are shared, in particular within the inactive (M4) and active intermediates (M3 and M5). In contrast, others such as those at the EC region of the G-protein competent state are unique. Taken together, this data suggests that cholesterol could bind to discrete sites of the receptor according to the state visited, possibly playing a role in the stabilization of the state itself.

## Discussion

By applying adaptive sampling combined with a goal-oriented scoring method, we were able to reconstruct the activation pathway(s) of the *apo* A2A receptor without *a priori* information of the active structure. We have shown that multiple active and inactive conformations could simultaneously coexist in a dynamic ensemble, which may account for the promiscuous coupling and signalling capabilities of GPCRs. Previously-solved agonist-bound crystal structures revealed the existence of A2A metastable intermediates along the path to activation. In our MSM model we have identified these intermediate states and fully characterized them. By comparing the obtained macrostates to solved A2A crystal structures in complex with ligands, we identified M0 and M4 as antagonist-competent states, M5 as the agonist-competent state and M2 as the G-protein competent state. Altogether, macrostates M0, M4 and M5 account for 70% of the overall *apo* population, thereby creating a pool of inactive and active intermediates to which ligands could bind. The existence of such a population of ligand-competent states in the *apo* A2A landscape supports Ye’s hypothesis^12^, that ligands bind to the receptor mainly *via* a conformational selection mechanism. However, in M5, residue Y197^5.58^ adopts a different orientation, pointing inward towards the helix bundle (in the crystal structure it points outwards). This crystal structure torsion was not observed in our analyses and suggests that subtle structural rearrangements could be induced by the ligand itself. Furthermore, the presence of conformations only partially similar to the solved miniG_s_-bound crystals hints at a scenario in which both conformational selection and induced fit could play a role in the binding of the G-protein. Indeed, it may help to explain why a single G-protein is able to bind many GPCRs, despite the fact that the G-protein binding site of GPCRs is poorly conserved across the family^51^. Of course, this would need to be further validated experimentally as crystallization artifacts may not be excluded.

Based on the generated MSM model, 66.2% of the total population of the *apo* receptor is shifted towards active-like intermediates such as M5, M3 and M2. Thus, we see an enrichment of active-like structures. However, it also appears that the *apo* receptor maintains a substantial percentage of its population (31.9%) in the inactive intermediate M4 state. The pathway analysis helps to understand the interplay between these states. M4 is highlighted as the conformational hub in the *apo* landscape and may enable activation by shifting towards both active intermediates (M3, M5). Macrostate M5, representing 38.4% of the population, is the state most prone to fast activation, thus it is primed to readily shift towards M2 upon agonist binding. Considering that M4 and M5 account for 70% of the total population, and that both can readily explore pathways that lead to full activation, these states essentially act as a conformational ‘reservoir’ for the receptor. This ‘reservoir’ would certainly prime A2A for activation when required (for example upon agonist binding), but also control the fraction of receptor amenable to basal activity (M2), scarcely populated in absence of G-protein^39^. Our final finding relates to the identification of cholesterol hotspots that are state-dependent. Recently, allosteric compounds have been widely exploited to modulate homologues of A2A^52^. In Fig. 5A we show an example of a cholesterol hotspot, for the inactive M4, that superimposes onto the allosteric modulator BPTU. It is difficult to assess the hotspots corresponding to M2, M3 and M5 due to the sparsity of lipid and allosteric modulators found to bind A2A in the corresponding conformations. These results are certainly intriguing and would require further study to clarify the potential for ligand regulation at these sites, opening new avenues for the allosteric modulation of A2A. To conclude, we have shown how activation in A2A is finely tuned and it is achieved, in a stepwise manner, thanks to the dynamic interplay among receptor conformational states. An interplay that is strongly influenced by binding of ligands and even of membrane lipids, such as cholesterol. Reconstruction of the activation pathways of the *apo* receptor has enabled a deeper understanding of its regulatory mechanisms with potential implications for drug discovery and allosteric regulation.

## Material and Methods

### System setup for MD simulations

The A2A inactive crystal structure (pdb code 5uig^31^) was used to perform the simulations described. The 5uig structure was first edited to remove BRIL and a model was constructed by modelling in the extracellular loop 2 (ECL2) residues (146–165) and the C-terminal aspect (from Phe295 onwards) of the A2A structure 4eiy using Prime(52). These steps were necessary as the ECL2 residues were not well defined in the 5uig structure and the C-terminal segment from Phe295 onwards appeared to be perturbed in 5uig. The absent intracellular loop 3 (ICL3) of the 5uig structure was modelled using the MEDELLER protocol(53), the core of which is a membrane-protein-specific version of PyFREAD for fragment-based loop modelling(54), with missing sidechains modelled using SCWRL3(55) and clashes removed using MODELLER(56). The template for the missing loop was the ICL3 loop taken from the A2A structure 3vg9(57). The structure was then prepared for simulation using the HTMD software^27^ wherein the co-crystallized ligand was deleted. Residue D52^2.50^ was protonated because important for GPCR activation and a sodium ion was placed as in crystal 4eiy because seen in inactive structures^47^. As expected, the sodium ion soon became unstable and egressed. Subsequently the *apo* protein was simulated using the CHARMM36 force field^53^ within a pre-equilibrated 80 × 80 POPC bilayer, supplemented with 20% cholesterol. The system was solvated with TIP3P water molecules, then Na+ and Cl− ions were added to obtain an ionic strength of 0.15 M. All-atom unbiased MD simulations were carried out using the ACEMD program^54^ running on GPUs, using a time-step of 4 fs with a hydrogen mass repartitioning scheme. The system was minimized with 500 steps of conjugated gradient followed, by 100 ns of NPT equilibration, employing a Berendsen barostat at 1 atm. The temperature was kept at 300 K using a Langevin thermostat. Heavy atoms of protein and lipids were constrained by employing a 1 kcal/mol/Å^2^ spring constant and they were gradually released during the equilibration. The Ewald algorithm was used for long-range electrostatic interactions with a 9 Å cutoff. All-atom production runs were performed using the AdaptiveGoal sampling protocol implemented in HTMD^27^. Additional details are described in SI Appendix.

### AdaptiveGoal sampling setup

All-atom production runs were performed using the AdaptiveGoal sampling protocol implemented in HTMD. A total of 1611 trajectories of 50 ns each were carried out on a dedicated GPU cluster equipped with Nvidia GeForce GTX1080 cards. Simulations of the *apo* A2A structure were carried out in the canonical (NVT) ensemble for an aggregated time of 80 μs. The adaptive sampling protocol allows an efficient exploration without adding any bias to the system, by performing simulations in successive epochs. A generic metric which considers α-Cα protein contact maps with a 5 Å threshold was associated to a more specific goal-oriented metric^30^. This latter metric considers a reduced number of dimensions relevant to GPCR activation (‘exploitation part’) namely: (i) the distance between the center of mass of R^3.50^ and E^6.34^; and (ii) the RMSD to inactive of the Y^7.53^. The ensemble of generated trajectories was then analyzed at each epoch by means of MSM, and starting conformations for the following epoch were identified using frames meeting the requirements of the goal function.

### MSM generation

The conformational space corresponding to the two chosen dimensions described above was discretized into 890 clusters using the mini batch k-means algorithm^55^. Subsequently, the clusters were clustered into 6 macrostates using the PCCA algorithm^56^, and the MSM was constructed using a 20 ns lag time. The converged timescales of the obtained MSM model can be seen in Fig. S1.

### G-protein binding site analysis

In order to evaluate the conformational changes towards the cytosolic aspect, we monitored the distances between the last cytosolic residues of transmembrane domains. In detail, we computed the distances between the residues of the TM5 and TM6 to all the others (TM 1 2 3 4 7) for a total of 10 distances. The residues selected were: M211(TM5), T224(TM6), V31(TM1), V40(TM2), R107(TM3), G118(TM4) and R291(TM7) (see Fig. S3 in Supporting Information).

We computed and summed these distances to have one value for each frame. We then computed the minimum, the maximum and the average value for each macrostate.

### Cholesterol analysis

To assess the cholesterol behaviour between the inactive and active conformations, we inspected the occupancy of the cholesterol molecules, with the aim to identify any hotspots around the GPCR transmembrane domain. First, we generated a trajectory for each macrostate by assigning each frame based on their macrostate membership. Thus, for each of these trajectories we identified the most probable cholesterol hotspots.

To identify these regions around the protein, we calculated a 3D histogram of 1 Å cubic bins of the cholesterol geometric center (using the MD simulations). From all these simulations we computed a count matrix of cholesterol centres of mass. These values were divided by the number of MD frames to retrieve the probability for each cubic grid. The probabilities were transformed into free energies with the Boltzmann equation:

$${\Delta }G={K}_{b}Tln(\frac{N}{{N}_{0}})$$

where T = temperature (300 K); K_B_ = Boltzmann constant(kcal/mol·K); N = cholesterol occupancy probability; N_0_ = cholesterol standard occupancy in equilibrium:

$${N}_{0}=\frac{{V}_{B}{N}_{A}[C]}{{\eta }_{B}}$$

Where V_B_ is the simulation box volume (liters); N_A_ the Avogadro’s number; C is the concentration (mol/L); η_B_ the number of boxes in the grid.

Minima closer than 8 Å were combined and the free energy of the clustered minima recomputed as the sum of the probabilities.

Finally, we kept only those hotspots that have an energy lower than −0.22 kcal/mol. See Fig. S4 in the Supplementary for more details.

## Supplementary information

Supporting Information. Reconstruction of apo A2A receptor activation pathways reveal ligand-competent intermediates and state-dependent cholesterol hotspots

## Data Availability

The datasets generated during and/or analysed during the current study are available from the corresponding author on reasonable request.
