# Supplementary material for: Reconstruction of apo A2A receptor activation pathways reveal ligand-competent intermediates and state-dependent cholesterol hotspots
Source: Sci Rep. 2019 Oct 2;9:14199. doi: 10.1038/s41598-019-50752-6 (PMC6775061; doi:10.1038/s41598-019-50752-6)
Supplement: Supplementary file 1 — Supporting Information. Reconstruction of apo A2A receptor activation pathways reveal ligand-competent intermediates and state-dependent cholesterol hotspots [file 41598_2019_50752_MOESM1_ESM.pdf]

Supporting Information

Reconstruction of *apo* A2A receptor activation pathways reveal ligand-competent intermediates and state-dependent cholesterol hotspots.

Silvia Lovera[1*], Alberto Cuzzolin[2], Sebastian Kelm[3], Gianni De Fabritiis[2,4,5], Zara A. Sands[1*]

1 CADD, UCB BioPharma, 1420 Braine l'Alleud, Belgium.
2 Acellera, Barcelona Biomedical Research Park (PRBB), C/Doctor Aiguader 88, 08003, Barcelona, Spain.
3 CADD, UCB Pharma, Slough, UK.
4 Computational Science Laboratory (GRIB-IMIM), Universitat Pompeu Fabra, Barcelona Biomedical Research Park (PRBB), C/Doctor Aiguader 88, 08003 Barcelona, Spain.
5 Institució Catalana de Recerca i Estudis Avançats (ICREA), Passeig Lluis Companys 23, Barcelona 08010, Spain.
* Correspondence: zara.sands@ucb.com, silvia.lovera@ucb.com

Supplementary Images

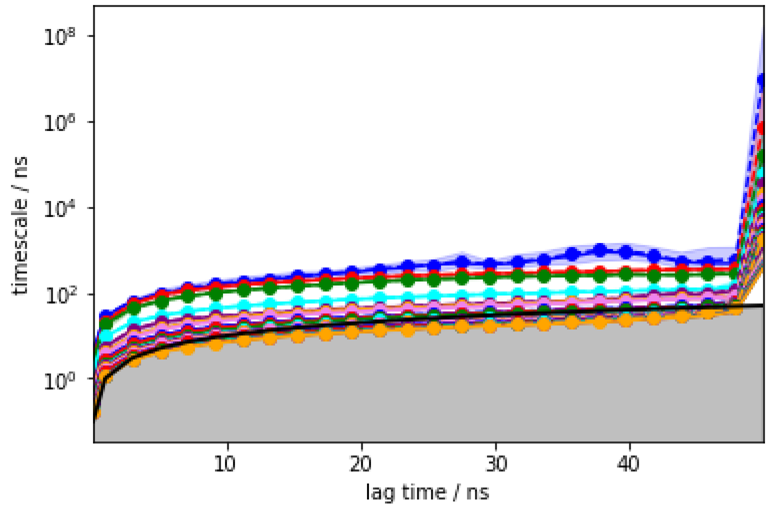

Figure S1: Implied timescale plot of the generated model.

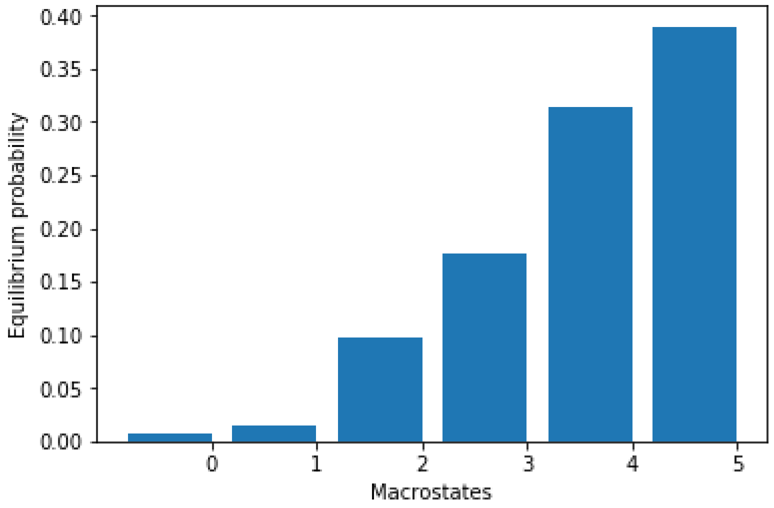

Figure S2: Equilibrium distribution plot of the *apo* A2A receptor obtained for the generated MSM model. Six kinetic macrostates were extracted. The respective equilibrium probabilities are as follows: M5=38.4% ± 2.3, M4=31.9% ± 1.5, M3=17.8% ± 1.3, M2=9.9% ± 1.1, M1=1.2% ± 0.4 and M0=0.6% ± 0.2.

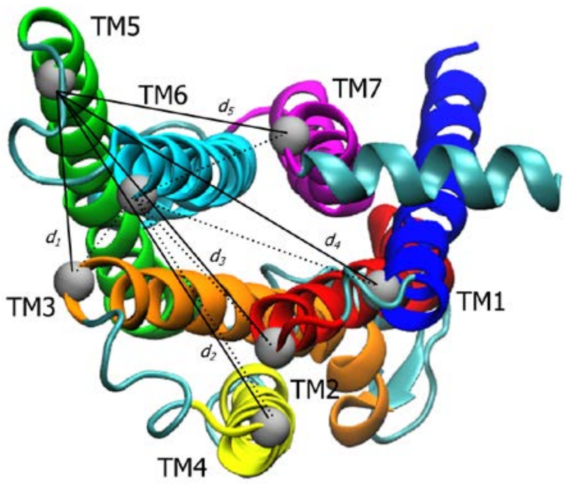

Figure S3: Intracellular portion of the A2A receptor. View of the distances used to calculate the G-protein binding site cavity. TM helices are coloured differently and the grey spheres corresponds to the CA atoms of the residues considered in the analysis.

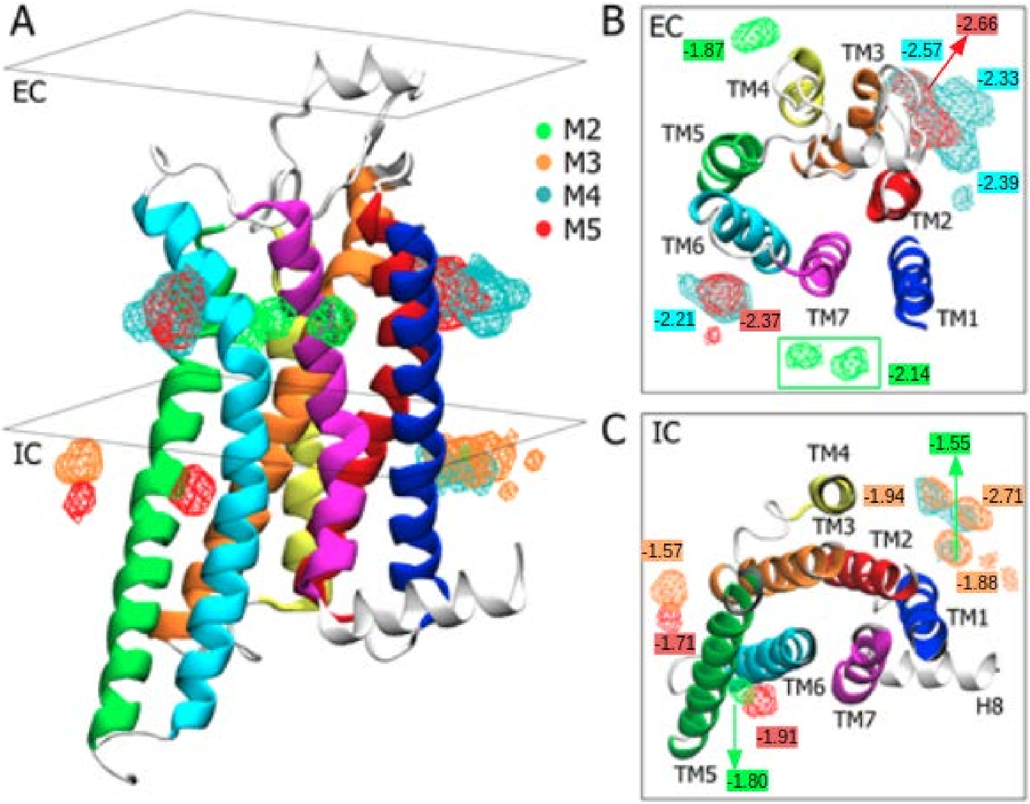

Figure S4: Cartoon representation of the A2A receptor showing cholesterol occupancy for each of the most populated macrostates: M2, M3, M4 and M5. (A) Mesh surfaces represent the hotspots where cholesterol has the higher occupancy. Each surface is colour-coded to correspond to the respective macrostate. (B) Transverse sectional view of the EC aspect of the A2A receptor. The hotspots corresponding to M2, M4 and M5 are shown in green, cyan and red respectively, along with their free energies (Kcal/mol). (C) Transverse sectional view of the IC aspect of the A2A receptor. The hotspots corresponding to M2, M3, M4 and M5 are shown in green, orange, cyan and red respectively, along with their free energies (Kcal/mol).

Supplementary Tables

Table S1: Summary of the aggregated simulation time and methodology used in recent published works of A2A and of other GPCRs. The list of published computational works on GPCRs, other than A2A, is not exhaustive. Here we considered the most renown studies in the field.

Study Ref	GPCR	Aggregated simulated time	Methodology	Notes
(Caliman et al., 2015)	A2A	3.5 μs	Classical MD	Deactivation pathway starting from both active and inactive
(Li et al., 2013)	A2A	12*10 μs	Classical MD Metadynamics	Exploration of *apo*, agonists-bound, antagonists-bound, G_s alpha5-bound
(Ng et al., 2013)	A2A	2*200 ns	Classical MD	Local exploration of *apo* and antagonist-bound
(Rodríguez et al., 2011)	A2A	8*100 ns	Classical MD	Local exploration of *apo* and antagonist-bound
(Dror et al., 2011)	β2AR	76*ranging from 2 to 50 μs	Classical MD	Deactivation from agonist-bound structure
(Kohlhoff et al., 2014)	β2AR	2.15 ms	Classical MD + MSM	Deactivation pathway starting from both active and inactive

(Shamsi et al., 2017)	β2AR	50 μs	Adaptive Monte Carlo using evolutionary coupling information	Activation pathway starting from inactive
(Miao et al., 2013)	M2	1.2 μs	Accelerated MD	Activation pathway starting from inactive
(Kapoor et al., 2017)	μ-opioid	2*240 μs	Adaptive MD	Exploration of agonist-bound structures

Table S2: Collection of the pdb crystal structures considered in this study and represented in Figure 2, panel A. Each number in Figure 2 corresponds to the pdb code reported in this table, followed by its publication reference and additional details, such as: name of the ligand bound and receptor conformation.

Assigned number in plot of Figure 2, panel A	PDB ID	Ref	Ligand	Conformation	Notes
0	3eml	(Jaakola et al., 2008)	ZMA	inactive	
1	5g53	(Carpenter et al., 2016)	NECA	fully active	with miniG$_s$
2	4eiy	(Liu et al., 2012)	ZMA	inactive	
3	2ydv	(Lebon et al., 2011)	NECA	active	
4	5uig	(Sun et al., 2017)	AT	inactive	
5	3qak	(Xu et al., 2011)	UK432097	active	
6	5nm2	(Weinert et al., 2017)	ZMA	inactive	
7	3pwh	(Doré et al., 2011)	ZMA	inactive	
8	3rfm	(Doré et al., 2011)	Caffeine	inactive	
9	4ug2	(Lebon et al., 2015)	NGI	active	

10	6dgd	(García-Nafría et al., 2018)	NECA	fully active	with miniG$_s$
11	5wf5	(White et al., 2018)	UKA	active	
12	2ydo	(Lebon et al., 2011)	Adenosine	active	
13	5olg	(Rucktooa et al., 2018)	ZMA	inactive	

References

Caliman, A.D., Swift, S.E., Wang, Y., Miao, Y., and McCammon, J.A. (2015). Investigation of the conformational dynamics of the apo A2A adenosine receptor. Protein Sci. *24*, 1004–1012.

Carpenter, B., Nehmé, R., Warne, T., Leslie, A.G.W., and Tate, C.G. (2016). Structure of the adenosine A(2A) receptor bound to an engineered G protein. Nature *536*, 104–107.

Doré, A.S., Robertson, N., Errey, J.C., Ng, I., Hollenstein, K., Tehan, B., Hurrell, E., Bennett, K., Congreve, M., Magnani, F., et al. (2011). Structure of the adenosine A(2A) receptor in complex with ZM241385 and the xanthines XAC and caffeine. Structure *19*, 1283–1293.

Dror, R.O., Arlow, D.H., Maragakis, P., Mildorf, T.J., Pan, A.C., Xu, H., Borhani, D.W., and Shaw, D.E. (2011). Activation mechanism of the β2-adrenergic receptor. Proc Natl Acad Sci USA *108*, 18684–18689.

García-Nafría, J., Lee, Y., Bai, X., Carpenter, B., and Tate, C.G. (2018). Cryo-EM structure of the adenosine A2A receptor coupled to an engineered heterotrimeric G protein. Elife *7*.

Jaakola, V.-P., Griffith, M.T., Hanson, M.A., Cherezov, V., Chien, E.Y.T., Lane, J.R., Ijzerman, A.P., and Stevens, R.C. (2008). The 2.6 angstrom crystal structure of a human A2A adenosine receptor bound to an antagonist. Science *322*, 1211–1217.

Kapoor, A., Martinez-Rosell, G., Provasi, D., de Fabritiis, G., and Filizola, M. (2017). Dynamic and Kinetic Elements of µ-Opioid Receptor Functional Selectivity. Sci. Rep. *7*, 11255.

Kohlhoff, K.J., Shukla, D., Lawrenz, M., Bowman, G.R., Konerding, D.E., Belov, D., Altman, R.B., and Pande, V.S. (2014). Cloud-based simulations on Google Exacycle reveal ligand modulation of GPCR activation pathways. Nat. Chem. *6*, 15–21.

Lebon, G., Warne, T., Edwards, P.C., Bennett, K., Langmead, C.J., Leslie, A.G.W., and Tate, C.G. (2011). Agonist-bound adenosine A2A receptor structures reveal common features of GPCR activation. Nature *474*, 521–525.

Lebon, G., Edwards, P.C., Leslie, A.G.W., and Tate, C.G. (2015). Molecular determinants of CGS21680 binding to the human adenosine A2A receptor. Mol. Pharmacol. *87*, 907–915.

Li, J., Jonsson, A.L., Beuming, T., Shelley, J.C., and Voth, G.A. (2013). Ligand-dependent activation and deactivation of the human adenosine A(2A) receptor. J. Am. Chem. Soc. *135*, 8749–8759.

Liu, W., Chun, E., Thompson, A.A., Chubukov, P., Xu, F., Katritch, V., Han, G.W., Roth, C.B., Heitman, L.H., IJzerman, A.P., et al. (2012). Structural basis for allosteric regulation of GPCRs by sodium ions. Science *337*, 232–236.

Miao, Y., Nichols, S.E., Gasper, P.M., Metzger, V.T., and McCammon, J.A. (2013). Activation and dynamic network of the M2 muscarinic receptor. Proc Natl Acad Sci USA *110*, 10982–10987.

Ng, H.W., Laughton, C.A., and Doughty, S.W. (2013). Molecular dynamics simulations of the adenosine A2a receptor: structural stability, sampling, and convergence. J. Chem. Inf. Model.

53, 1168–1178.

Rodríguez, D., Piñeiro, Á., and Gutiérrez-de-Terán, H. (2011). Molecular dynamics simulations reveal insights into key structural elements of adenosine receptors. Biochemistry *50*, 4194–4208.

Rucktooa, P., Cheng, R.K.Y., Segala, E., Geng, T., Errey, J.C., Brown, G.A., Cooke, R.M., Marshall, F.H., and Doré, A.S. (2018). Towards high throughput GPCR crystallography: In Meso soaking of Adenosine A2A Receptor crystals. Sci. Rep. *8*, 41.

Shamsi, Z., Moffett, A.S., and Shukla, D. (2017). Enhanced unbiased sampling of protein dynamics using evolutionary coupling information. Sci. Rep. *7*, 12700.

Sun, B., Bachhawat, P., Chu, M.L.-H., Wood, M., Ceska, T., Sands, Z.A., Mercier, J., Lebon, F., Kobilka, T.S., and Kobilka, B.K. (2017). Crystal structure of the adenosine A2A receptor bound to an antagonist reveals a potential allosteric pocket. Proc Natl Acad Sci USA *114*, 2066–2071.

Weinert, T., Olieric, N., Cheng, R., Brünle, S., James, D., Ozerov, D., Gashi, D., Vera, L., Marsh, M., Jaeger, K., et al. (2017). Serial millisecond crystallography for routine room-temperature structure determination at synchrotrons. Nat. Commun. *8*, 542.

White, K.L., Eddy, M.T., Gao, Z.-G., Han, G.W., Lian, T., Deary, A., Patel, N., Jacobson, K.A., Katritch, V., and Stevens, R.C. (2018). Structural Connection between Activation Microswitch and Allosteric Sodium Site in GPCR Signaling. Structure *26*, 259–269.e5.

Xu, F., Wu, H., Katritch, V., Han, G.W., Jacobson, K.A., Gao, Z.-G., Cherezov, V., and Stevens, R.C. (2011). Structure of an agonist-bound human A2A adenosine receptor. Science *332*, 322–327.
